# Nicotine dependence is associated with an increased risk of developing chronic, non-communicable inflammatory disease: a large-scale retrospective cohort study

**DOI:** 10.3389/fpsyt.2025.1429297

**Published:** 2025-02-12

**Authors:** Khalaf Kridin, Cristian Papara, Katja Bieber, David A. De Luca, Jan Philipp Klein, Marlene A. Ludwig, Philip Curman, Artem Vorobyev, Astrid Dempfle, Ralf J. Ludwig

**Affiliations:** ^1^ Lübeck Institute of Experimental Dermatology, University of Lübeck, Lübeck, Germany; ^2^ Azrieli Faculty of Medicine, Bar-Ilan University, Safed, Israel; ^3^ Unit of Dermatology and Skin Research Laboratory, Galilee Medical Center, Nahariya, Israel; ^4^ Institure and Comprehensive Centre for Inflammation Medicine, University Hospital Schleswig-Holstein (UKSH), Lübeck, Germany; ^5^ Department of Psychiatry, Psychosomatics and Psychotherapy, Lübeck University, Lübeck, Germany; ^6^ Independent Researcher, Groß Grönau, Germany; ^7^ Dermato-Venereology Clinic, Karolinska University Hospital, Stockholm, Sweden; ^8^ Department of Medical Epidemiology and Biostatistics, Karolinska Institutet, Stockholm, Sweden; ^9^ Department of Medicine Solna, Karolinska Institutet, Stockholm, Sweden; ^10^ Department of Dermatology, University Hospital Schleswig-Holstein (UKSH), Lübeck, Germany; ^11^ Institute of Medical Informatics and Statistics, Kiel University, Kiel, Germany

**Keywords:** nicotine dependence, chronic inflammatory diseases, asthma, lupus, COPD, dermatomyositis, granulomatosis with polyangiitis (GPA), pyoderma gangrenosum

## Abstract

**Introduction:**

Chronic, non-communicable inflammatory diseases (CIDs) affect a large portion of the population, imposing a significant morbidity, encompassing a substantial mortality. Thus, they are a major medical burden with a high unmet need. CIDs develop over the span of several years, and the risk of developing CIDs has been linked to genetic and environmental factors. Thus, modification of environmental factors is a promising approach for the prevention of CIDs. Among modifiable environmental factors that have been linked to the CID risk is nicotine dependence. However, for only few CIDs, compelling evidence suggests that nicotine dependence increases (e.g., rheumatoid arthritis and asthma) or decreases (e.g., pemphigus) the CID risk. For most CIDs, there are inconsistent, scant, or no reports on the risk of CID associated with nicotine dependence.

**Methods:**

To address this gap, we leveraged TriNetX, analyzing data from over 120 million electronic health records (EHRs). Using propensity score matching (PSM) to control for age, sex, ethnicity, and other CID risk factors, we contrasted the risk of developing any or any of the 38 CIDs in 881,192 EHRs from individuals with nicotine dependence to PSM-matched unexposed counterparts.

**Results:**

The analytical pipeline was validated by demonstrating an increased risk of individuals exposed to nicotine dependence for subsequent diagnosis of myocardial infarction, malignant neoplasm of the lung, and chronic obstructive pulmonary disease. Overall, 16.8% of individuals with nicotine dependence developed CIDs, compared to 9.6% of individuals not exposed to nicotine dependence (hazard ratio 2.12, confidence interval 2.10–2.14, *p* < 0.0001). Investigating single CIDs, nicotine dependence imposed increased risks for 23 of the 38 investigated diseases, i.e., dermatomyositis, granulomatosis with polyangiitis, pyoderma gangrenosum, and immune thrombocytopenic purpura. The sex-stratified analysis revealed few sex-specific differences in CID risk.

**Discussion:**

Our study emphasizes the importance of preventive measures targeting nicotine addiction to reduce the global burden of CIDs.

## Introduction

Chronic, non-communicable inflammatory diseases (CIDs) are caused by uncontrolled activation of the immune system leading to tissue damage and functional impairment. Over the past decades, their prevalence has constantly increased, affecting approximately 20%–25% of the population. Despite increased insights into disease mechanisms, treatment of CID is mainly based on immunosuppression. Albeit this allows for a relatively good disease control, continued treatment is required, and relapses and/or flares are common (1, 2). Like other complex diseases, CIDs develop over the span of several years. This is best documented for systemic lupus erythematosus (SLE) and rheumatoid arthritis (RA), CIDs where autoantibodies are key pathogenic drivers. Autoantibodies in SLE and RA are present years before the onset of clinical symptoms, indicating that progression towards clinically manifest disease indeed spans over several years (3–5). Generally, in CIDs, disease develops in the context of a complex interplay between genetics and the environment (6–8). Given the lack of curative treatment options and the magnitude of morbidity imposed by CIDs, identifying modifiable risk factors for CIDs is a prerequisite for the implementation of preventive measures.

Nicotine dependence, especially smoking, has been suggested to be among potentially modifiable CID risk factors that may even imprint long-lasting health effects after short-term exposure (9). This assumption stems from observations in patients with SLE, RA, hidradenitis suppurativa, psoriasis, multiple sclerosis, chronic obstructive pulmonary disease (COPD), and asthma, where current and/or past nicotine dependence confers an increased risk for subsequent diagnosis of the aforementioned CIDs (8, 10–15). Conversely, nicotine dependence has also been demonstrated to be associated with a lower risk for certain CIDs, namely, pemphigus (16, 17). However, for most CIDs, the potential impact of nicotine dependence on subsequent disease manifestation is (i) reported inconsistently, (ii) less well established, or (iii) largely unknown.

In more detail, inconsistent results on the impact of nicotine dependence on CIDs have been reported for the following: In sarcoidosis, nicotine dependence has been reported to have no (18), a reduced (19), or an increased (20) risk for disease manifestation. In atopic dermatitis, no increased risk with current nicotine dependence was noted in a large cohort of US women (21). In contrast, previous studies had noted that nicotine dependence is associated with an increased atopic dermatitis prevalence (22, 23). Likewise, discrepant data have been reported for celiac disease (24, 25), Crohn’s disease, and ulcerative colitis (26). Furthermore, some reports indicated that symptoms of systemic sclerosis are aggravated by nicotine dependence (27), albeit other studies refuted this finding (28).

Regarding the following CIDs, only scant insights into the risk of nicotine dependence on CIDs have been reported: The risk of developing systemic sclerosis in nicotine dependence compared to a matched control group was similar (29). In a case–control study, incident ankylosing spondylitis was found to be associated with current nicotine dependence (30). In eosinophilic granulomatosis with polyangiitis (EGPA) and vitiligo (31), nicotine dependence was associated with decreased disease risks (32). An increased risk for disease manifestation in individuals with nicotine dependence was observed for alopecia areata (33), lichen sclerosus in men (34), primary biliary cirrhosis (PBC) (35), and autoimmune hepatitis (36). In autoimmune thyroiditis, smoking cessation is followed by a transient rise in the incidence of autoimmune hypothyroidism (37). In type 1 diabetes, maternal nicotine dependence reduced the risk of diabetes manifestation (38). Lastly, myasthenia gravis patients who had been smoking at the onset of disease were significantly younger compared with those who had never smoked. For the most part, these reports have not been validated (or refuted) in other studies.

No information on the risk of nicotine dependence on the following CIDs could be retrieved from PubMed with the search term (“nicotine dependence” OR smoking) “disease name”, performed in April 2023: Polymyalgia rheumatica, dermatomyositis, granulomatosis with polyangiitis (GPA), pyoderma gangrenosum, lichen planus, morphea/localized scleroderma, bullous pemphigoid (BP), mucous membrane pemphigoid (MMP), pernicious anemia, autoimmune hemolytic anemia, and immune thrombocytopenic purpura.

In addition to these knowledge gaps, sex-specific risks of nicotine dependence had only been determined in a minority of studies (39, 40). This thus warrants subgroup analysis stratified by sex.

To clarify the impact of nicotine dependence on the risk of CID, which includes a sex-stratified subgroup analysis, we used TriNetX, a large-scale, global database, encompassing over 120 electronic health records (EHRs). For this, we contrasted the risk of developing each or any of 38 CIDs in persons with current or past nicotine dependence (exposed individuals) to propensity-matched unexposed individuals. To validate the approach, we also determined the risk of myocardial infarction (MI), lung cancer, and COPD, where nicotine dependence confers a high risk of disease manifestation (14, 41, 42). These insights into the overall and sex-specific risks for CIDs imposed by nicotine dependence will provide solid evidence for implementation of preventive measures, especially in individuals at risk of developing CIDs.

## Materials and methods

### Study design and database

**Figure 1:**
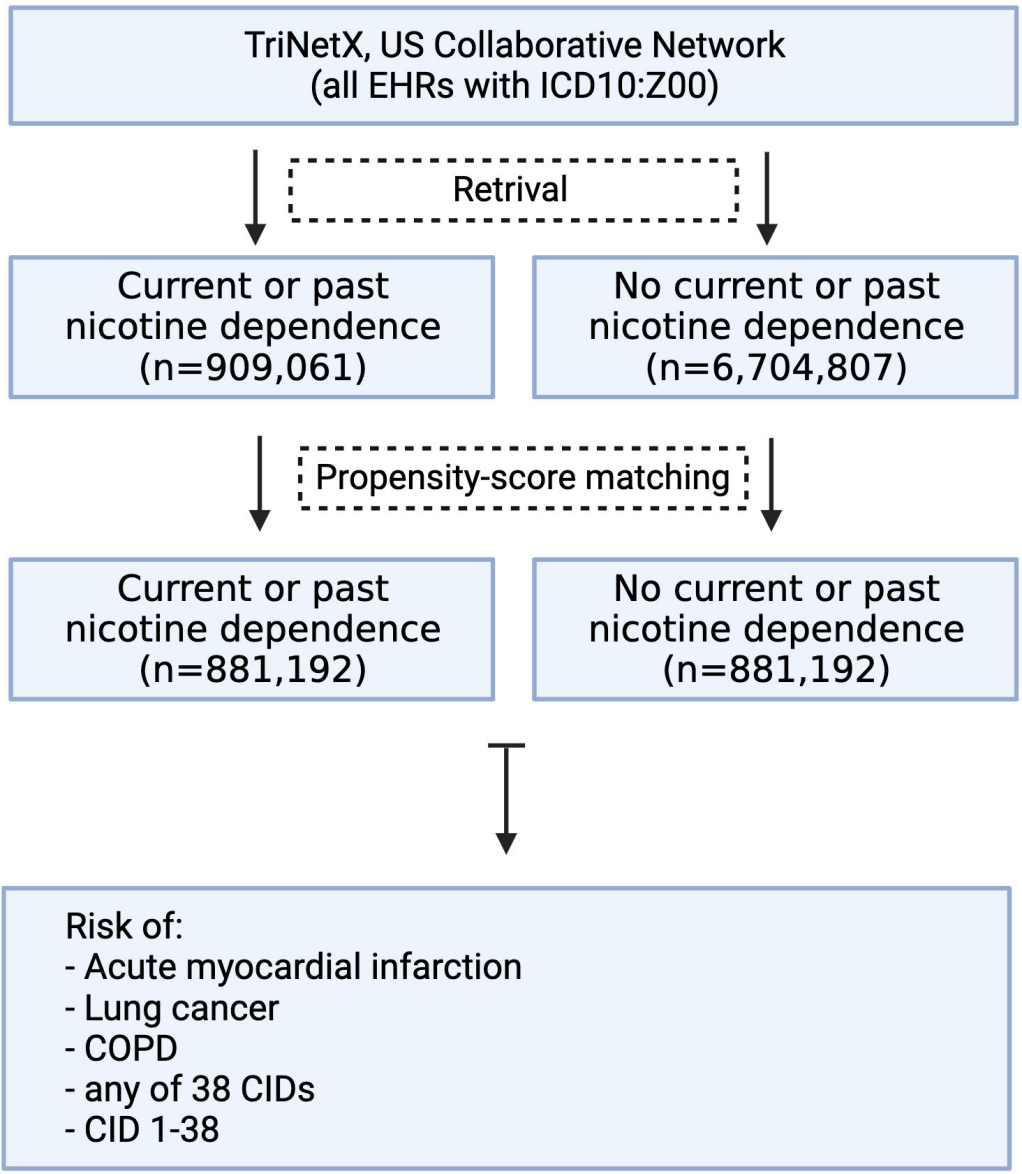
Study flowchart.

A global population-based retrospective cohort study with propensity score matching (PSM) was performed following previously published protocols (43–45). More specifically, we first retrieved EHRs with documentation of current or past nicotine dependence in those individuals presenting for “Encounter for general examination without complaint, suspected or reported diagnosis” (exposed group). Next, EHRs without a documentation of current or past nicotine dependence were retrieved from individuals presenting for “Encounter for general examination without complaint, suspected or reported diagnosis” (unexposed group). All EHRs were retrieved from the US Collaborative Network of TriNetX, which, at the time of analysis, included over 101 million EHRs from 60 healthcare organizations (HCOs). We then contrasted the risk of exposed and unexposed individuals of developing any or either one of a total of 38 chronic, non-communicable inflammatory diseases (CIDs, **Figure 1**). To validate the study design, we included three diseases, where nicotine dependence is a well-documented risk factor, specifically malignant neoplasms of the lung and bronchus, acute MI, and COPD. To address potential sex-specific differences, we performed a subgroup analysis for female and male patients, following the above-outlined procedures. Analyses were performed from September to October 2023. To account for unmeasured bias, we conducted a sensitivity analysis where nicotine dependence had to be documented at least 1 year apart. The sensitivity analysis was performed in December 2024. Based on a collaborative agreement of TriNetX and the UKSH, all UKSH employees have access to TriNetX.

### Ethics statement

Data accessible via TriNetX are presented in aggregate form and only contain anonymized data as per the de-identification standard defined by the US Health Insurance Portability and Accountability Act (HIPAA) in section §164,514(a). As this study exclusively used de-identified anonymized electronic medical records, it did not require Institutional Review Board approval.

### Study population and definition of eligible patients

Exposed individuals with current or past nicotine dependence were defined by the presence of ICD10:Z00 and ICD10:F17 or ICD10:Z87.891. ICD10:F17 “Nicotine dependence” includes ICD10:F17.20 “Nicotine dependence, unspecified”, ICD10:F17.21 “Nicotine dependence, cigarettes”, ICD10:F17.22 “Nicotine dependence, chewing tobacco”, and ICD10:F17.29 “Nicotine dependence, other tobacco product”. Of these, ICD10:F17.20 and F17:21 comprise over 95% of the total coding for ICD10:F17. ICD10:Z87.891 encodes for “Personal history of nicotine dependence”. Unexposed individuals were defined by ICD10:Z00 and the absence of ICD10:F17 and ICD10: Z87.891. Therefore, both exposed and unexposed groups were retrieved from individuals presenting for “Encounter for general examination without complaint, suspected or reported diagnosis” (ICD10:Z00). For subgroup analysis, the same exposed/unexposed groups were retrieved for female or male patients only, respectively. To ensure follow-up and validate the presence of nicotine dependence, inclusion and exclusion criteria had to be met twice with an interval of at least 3 months. In the sensitivity analysis, cases and controls were also retrieved from EHRs with ICD10:Z00. In the nicotine dependence exposed group, documentation of ICD10:F17 served as the index event. To be included in this group, ICD10:F17 had to be documented a second time, which occurred at least 12 months after its first documentation. To ensure follow-up in the controls, a visit occurring at least 12 months after the initial healthcare encounter had to be documented after the documentation of ICD10:Z00. To differentiate between smoking (encoded by ICD10:F17.21) and nicotine dependence (encoded by ICD10:F10 and ICD10:Z87.891), a validation analysis was performed. Here, the risks of malignant neoplasms of the lung, MI, COPD, any CID, and any CID without asthma and COPD were evaluated.

### Covariates

PSM was performed by establishing a covariate matrix including demographic information and known risk factors of CIDs. Covariates included were as follows: age at index (continuous variable), female gender (binary), White ethnicity (binary), the presence/absence of overweight and obesity (ICD10:E66), the presence/absence of a family history of other diseases of the musculoskeletal system and connective tissue (ICD10:Z82.69), the presence/absence of reaction to severe stress and adjustment disorders (ICD10:F43), and the presence/absence of problems related to life management difficulty (ICD10:Z73). The latter two related to stress have been selected to best reflect “stress” by using ICD10 codes. The matrix row order was randomized after data retrieval. A propensity score for each patient was generated by logistic regression analysis (with exposure as the dependent variable) using the Python package scikit-learn. Matching was performed 1:1 using the greedy nearest-neighbor approach with a cutoff distance of 0.1 pooled standard deviations of the logit of the propensity score. Baseline characteristics were re-evaluated and reported after matching, and differences were compared by *t*-test for continuous and *z*-test for binary or categorical variables.

### Power and statistical analysis

With an available retrospective matched cohort study of 881,192 exposed individuals, the same number of unexposed individuals and an adjusted significance level of alpha = 0.05/38 = 0.0013, we will have more than 80% power to detect HRs of at least 1.7, assuming a CID incidence of 0.01% in unexposed individuals and HRs of at least 1.2 for a CID incidence of 0.1% in unexposed individuals. For statistical analysis, the index event was set as the diagnosis of nicotine dependence or a history thereof, or the reported healthcare encounter in the unexposed group, respectively. CID diagnoses at any time after the index event were considered in the analysis. CID diagnoses prior to the diagnosis of each index event were excluded. Relative risks and risk differences were calculated. Survival analyses were performed using the Kaplan–Meier (KM) method. KM curves were compared using the log-rank test; *p*-values of less than 0.05/38 = 0.0013 were considered significant (Bonferroni correction). Nelson–Aalen plots were utilized to investigate the proportionality assumption. A univariate Cox proportional hazards regression was used to calculate hazard ratios (HRs). All reported confidence intervals (CIs) are 95% CIs.

### Use of AI and AI-assisted technologies in scientific writing

ChatGPT 3.5 (https://chat.openai.com/?model=text-davinci-002-render-sha) was used in the writing process to improve readability. Suggestions made by AI were critically reviewed and modified if applicable.

## Results

### Validation of study design: nicotine dependence confers an increased risk for malignant neoplasms of the lung, myocardial infarction, and COPD

**Figure 2:**
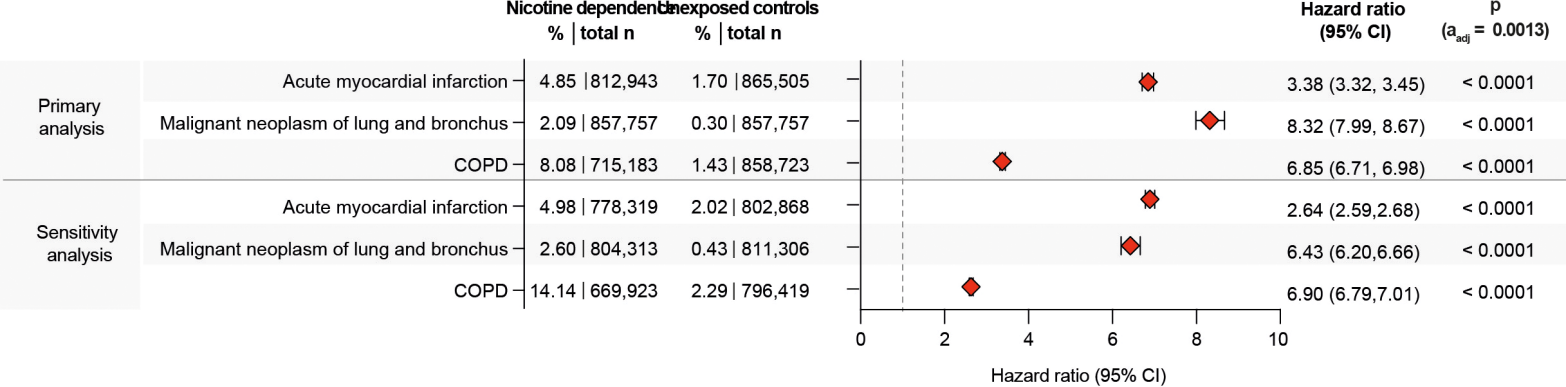
Nicotine dependence increases the risk of developing chronic obstructive pulmonary disease, lung cancer, and acute myocardial infarction. For validation, the risk for chronic obstructive pulmonary disease (COPD), lung cancer, and acute myocardial infarction was compared between cases and controls. As expected, the risk for all three diseases was increased in persons with documented current or past nicotine dependence as opposed to those without.

**Figure 3:**
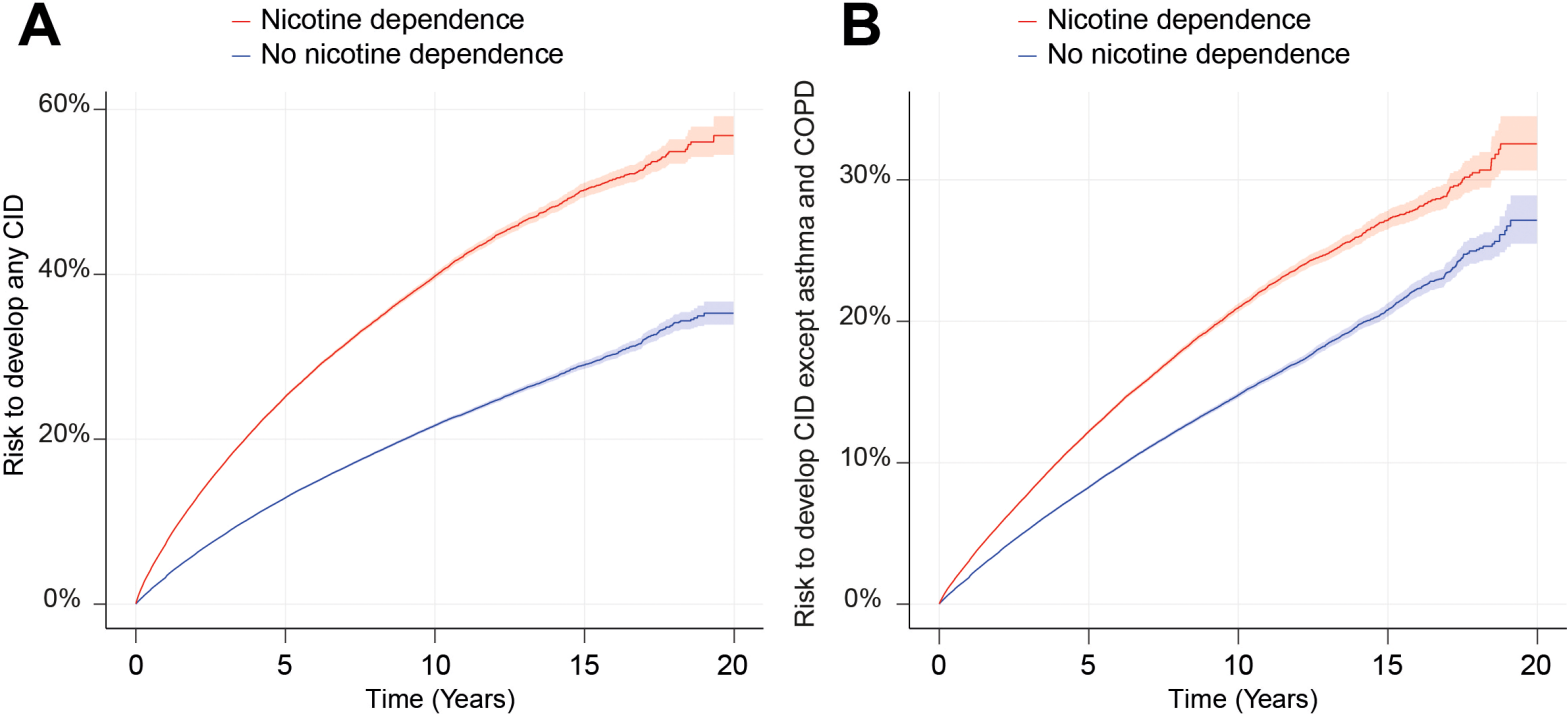
Nicotine dependence increases the risk of developing chronic, non-communicable inflammatory diseases (CIDs). We compared the risk of persons with documented current or past nicotine dependence (exposed) of developing any or any one of 38 CIDs to that of persons without documented current or past nicotine dependence in the US Collaborative Network of TriNetX. **(A)** The Nelson–Aalen plot of the risk of developing any of the 38 selected CIDs in persons with current or past nicotine dependence (red) compared to those without (blue). The shaded line indicates standard error. **(B)** The Nelson–Aalen plot of the risk of developing any of the 38 selected CIDs, except for asthma and COPD in persons with current or past nicotine dependence (red) compared to those without (blue). The shaded line indicates standard error.

**Figure 4:**
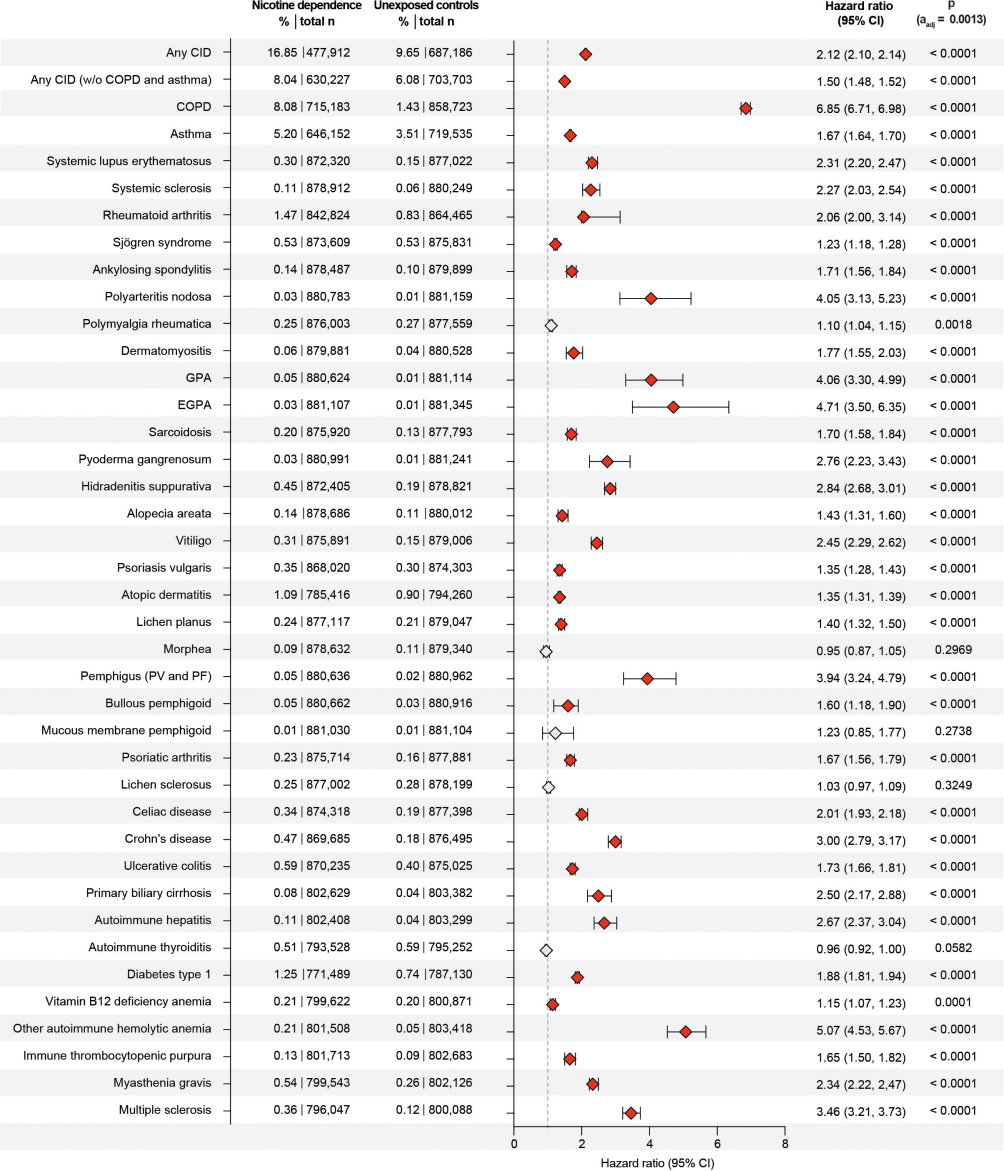
Nicotine dependence increases the risk of developing chronic, non-communicable inflammatory diseases (CIDs). We compared the risk of persons with documented current or past nicotine dependence (exposed) of developing any or any one of 38 CIDs to that of persons without documented current or past nicotine dependence in the US Collaborative Network of TriNetX. Exposed individuals (with a history of current or past nicotine dependence) were matched 1:1 to unexposed individuals (without any documentation of current of past nicotine dependence at the index healthcare visit) using age, sex, ethnicity, and risk factors of chronic, non-communicable inflammatory diseases. Non-significant data (after adjustment for multiple testing) is indicated by light gray. Hazard ratios were calculated by univariate Cox regression *p*-values determined by the Log-rank test. COPD, chronic obstructive pulmonary disease; GPA, granulomatosis with polyangiitis; EGPA, eosinophilic granulomatosis with polyangiitis; PV, pemphigus vulgaris; PF, pemphigus foliaceus.

After propensity matching, 881,192 EHRs were obtained for both exposed and unexposed individuals. Cohorts exhibited slight yet significant variations in age, ethnicity, and sex distribution, with age and the proportion of female patients showing the most notable differences (**Table 1**). After retrieval of cohorts, we first aimed to validate our approach within this cohort by focusing on three diseases where nicotine dependence serves as a well-established risk factor: COPD (14), lung cancer (46), and acute MI (47). Our study reaffirms prior findings, indicating a heightened risk for COPD (HR 6.85, CI 6.71–6.98, *p* < 0.0001), lung cancer (HR 8.32, CI 7.99–8.67, *p* < 0.0001), and MI (HR 3.38, CI 3.32–3.45, *p* < 0.0001) among individuals with current or past nicotine dependence compared to those without any history of nicotine dependence (**Figure 2**). These findings persisted in sensitivity analysis, mandating a documentation of nicotine dependence at two instances at least 1 year apart (**Figures 2**–**4**, **Table 2**). Furthermore, no major differences in these risks were observed in the validation experiments, which compared the risks among individuals with nicotine dependence (versus their respective controls) to those among individuals exposed to smoking (versus their respective controls, **Supplementary Table 1**).

**Table 1:** Baseline characteristics before and after propensity score matching of electronic health records (EHRs) indicating current or past nicotine dependence (exposed) and those without the documentation of current of past nicotine dependence (unexposed).

Characteristic	Before matching			After matching		
	Nicotine dependence (exposed)	Unexposed	*p*-value	Nicotine dependence (exposed)	Unexposed	*p*-value
Number of participants	909,061	6,704,807	–	881,192	881,192	–
Age at index (years, SD)	55.0 ± 19.7	29.7 ± 25.9	<0.001	54.8 ± 19.8	55.0 ± 19.9	<0.001
Female (%)	51.9	54.4	<0.001	51.7	52.2	<0.001
White (%)	71.7	60.1	<0.001	71.4	71.2	0.028
Overweight and obesity	34.7	10.8	<0.001	33.5	33.4	0.111
Family history of other diseases of the musculoskeletal system and connective tissue	0.3	0.1	<0.001	0.2	0.2	0.013
Reaction to severe stress and adjustment disorders	11.2	2.6	<0.001	10.2	10.9	<0.001
Problems related to life management difficulty	4.2	0.2	<0.001	1.9	1.4	<0.001

**Table 2:** Baseline characteristics before and after propensity score matching of electronic health records (EHRs) indicating current or past nicotine dependence (exposed) and those without the documentation of current of past nicotine dependence (unexposed) for sensitivity analysis 1.

Characteristic	Before matching			After matching		
	Nicotine dependence (exposed)	Unexposed	*p*-value	Nicotine dependence (exposed)	Unexposed	*p*-value
Number of participants	825,435	12,496,130	–	813,204	813,204	–
Age at index (years, SD)	50.4 ± 15.1	34.9 ± 25.6	<0.0001	50.4 ± 15.1	50.4 ± 15.1	0.8089
Female (%)	46.7	52.923	<0.0001	46.7	46.706	0.9336
White (%)	66.009	60.775	<0.0001	66.009	66.0	0.8973
Overweight and obesity	20.303	6.779	<0.0001	20.303	20.315	0.8378
Family history of other diseases of the musculoskeletal system and connective tissue	0.095	0.031	<0.0001	0.095	0.092	0.5547
Reaction to severe stress and adjustment disorders	9.055	1.658	<0.0001	9.054	9.069	0.7534
Problems related to life management difficulty	0.296	0.06	<0.0001	0.095	0.092	<0.0001

Note that covariates highlighted in gray were not used for PSM because the combined sample size of both cohorts only allows to include five covariates for PSM.

### Nicotine dependence is associated with an increased risk for the development of any CID

We compared the risk of developing any of the selected 38 CIDs, including COPD, between individuals with current or past nicotine dependence and those without. Among non-nicotine-exposed individuals, the risk of developing any of the 38 CIDs was 9.65%, which increased to 16.85% in individuals exposed to nicotine dependence. This marked difference resulted in a hazard ratio of 2.12 (CI 2.10–2.14, *p* < 0.0001, **Figures 3** and **4**). As COPD and asthma notably influenced the disparity between exposed and non-exposed individuals, we conducted a secondary analysis excluding these conditions to isolate the impact of nicotine dependence on CIDs other than COPD and asthma. The risk of developing any of the remaining 36 CIDs was 6.08% in unexposed individuals, rising to 8.04% in those exposed to nicotine dependence. Once again, this led to a significant increase in CID development associated with nicotine dependence (HR 1.50, CI 1.48–1.52, *p* < 0.0001, **Figures 3**, **4**). Again, these findings remained consistent in sensitivity analysis, mandating a documentation of nicotine dependence at two instances at least 1 year apart (**Figures 3**, **4**, **Table 2**; **Supplementary Figure 1**). Furthermore, no major differences in these risks were observed in the validation experiments, which compared the risks among individuals with nicotine dependence (versus their respective controls) to those among individuals exposed to smoking (versus their respective controls, **Supplementary Table 1**).

### 23 At the individual CID level, nicotine dependence confers an increased risk for out of 38 CIDs, has no impact on the risk for 9 out of 38 CIDs, and leaves uncertainty for six diseases

The assessment of specific CIDs in the initial analysis revealed that current or past nicotine dependence heightened the risk for 33 out of 38 CIDs, with no discernible impact on the risk for the remaining 5 (**Figures 3**, **4**). Notably, the most substantial risk increase was observed for COPD (HR 6.85, CI 6.71–6.89, *p* < 0.0001), where individuals with current or past nicotine dependence exhibited an 8.08% risk of incident COPD diagnosis compared to 1.43% in those without nicotine exposure. Additionally, nicotine dependence correlated with a 5.20% risk of subsequent asthma diagnosis, in contrast to 3.51% in non-exposed individuals, resulting in an HR of 1.67 (CI 1.64–1.70, *p* < 0.0001). Moreover, nicotine dependence was associated with an elevated risk for future diagnoses of granulomatosis with polyangiitis (GPA, HR 4.06, CI 3.30–4.99, *p* < 0.0001). Nicotine dependence also increased the risk for several diseases with HRs equal to or greater than 2.5, including hidradenitis suppurativa, pemphigus, Crohn’s disease, PBC, autoimmune hepatitis, autoimmune hemolytic anemia, and multiple sclerosis. These findings persisted in sensitivity analysis mandating documentation of prolonged nicotine dependence (**Figures 3**, **4**; **Supplementary Figure 1**). For polymyalgia rheumatica and mucous membrane pemphigoid, none of the analyses showed an impact of nicotine dependence of CID risk. Increased risks for the following CIDs were observed in the primary but not replicated in the sensitivity analysis: systemic sclerosis, polyarteritis nodosa, EGPA, alopecia areata, lichen planus, pemphigus, and bullous pemphigoid. Otherwise, discordant results were observed for Sjögren syndrome, vitiligo, localized scleroderma (morphea), lichen sclerosus, celiac disease, and autoimmune thyroiditis (**Figures 3**, **4**, **Table 2**; **Supplementary Figure 1**). Taken together, the results establish nicotine dependence as a risk factor for 23 CIDs, exclude nicotine dependence as a risk factor for 9 CIDs, and leave uncertainly on the risk for six CIDs.

### Few sex-specific CID risks imposed by exposure to nicotine dependence

Given the disparities in sex distribution (**Table 1**), and to discern potential sex-specific risks, we conducted subgroup analyses stratified by female or male sex. In the analysis confined to EHRs with female sex, an elevated risk of developing 33 out of 38 CIDs was observed. For 2 of the 38 CIDs, a decreased risk was noted following exposure to nicotine, while in 3 of the 38 CIDs, no discernible impact of nicotine dependence on CID risk was observed. Specifically, consistent with the findings from the non-sex-stratified analysis, nicotine dependence was associated with an increased risk for subsequent development of any CID (HR 1.91, CI 1.87–1.94, *p* < 0.0001, **Supplementary Tables 1**, **2**). Comprehensive details on all CIDs with an increased risk following exposure to nicotine dependence and demographic information for the female-stratified analysis are provided in **Supplementary Tables 2**, **3**. Exposure to nicotine dependence had no significant impact on the risk of developing polymyalgia rheumatica [HR 1.10, CI 1.01–1.19, *p* = 0.0245 (α_adj_. = 0.0013)], MMP (HR 1.46, CI 0.88–2.41, *p* = 0.1404), and lichen sclerosus (HR 0.96, CI 0.90–1.02, *p* = 0.1504). However, in contrast to the unstratified analysis, a decreased risk was noted for morphea (HR 0.77, CI 0.69–0.86, *p* < 0.0001), and autoimmune thyroiditis (HR 0.92, CI 0.88–0.97, *p* = 0.0008) in the analysis focused on female patients.

In the analysis including only EHRs with male sex, 373,006 EHRs were retrieved for both cases and controls (**Supplementary Table 4**). Subsequent development of any CID was noted in 14.16% of those exposed to nicotine dependence, as opposed to 7.58% in the non-exposed controls. This difference translates into an HR of 2.22 (CI 2.18–2.26, *p* < 0.0001, **Supplementary Table 5**). Again, most of this risk increment was observed for COPD and asthma. Excluding these two diagnoses, the risk of developing any of the “remaining” CIDs remained elevated in the group characterized by nicotine dependence (HR 1.66, CI 1.62–1.69, *p* < 0.0001). Specifically, it was observed for 30 out of 38 CIDs. For none of the 38 CIDs, a decreased risk was noted following exposure to nicotine, while in 8 of the 38 CIDs, no discernible impact of nicotine dependence on CID risk was observed (**Supplementary Tables 4**, **5**).

## Discussion

We here obtained comprehensive insights into the risk for CIDs imposed by nicotine dependence. Our data suggest that nicotine dependence imposes an increased risk for the manifestation of CIDs. Thus, nicotine dependence is a modifiable risk factor for the primary prevention of CIDs.

Foremost, the data clarify the previously uncertain impact of nicotine dependence in several CIDs. More specifically, in sarcoidosis, nicotine dependence had been reported to have no (18), a reduced (19), or an increased (20) risk for disease manifestation. We here now provide solid evidence that nicotine dependence is associated with an increased risk for sarcoidosis. This risk increment was observed when controlling analysis for age, sex, race, and ethnicity, as well as CID risk factors. Likewise, in atopic dermatitis, studies indicated that nicotine dependence is associated with an increased atopic dermatitis prevalence (22, 23). In contrast, no increased risk with current nicotine dependence was noted in a large cohort of US women (21). The results obtained herein demonstrate that nicotine dependence is associated with an increased risk for atopic dermatitis. This risk increment for atopic dermatitis in individuals with current or past nicotine dependence is also observed in the analyses stratified for female or male sex. These differences may be explained by differences in study design: In the meta-analysis of Kantor and colleagues, no adjustment for potential confounding factors was made (22). We here considered stress and obesity as potential confounding factors and included these in the propensity matching. Likewise, in the study by Lee and colleagues, which also demonstrated an increased risk for atopic dermatitis in persons with nicotine dependence, these factors were also not considered (23). However, both obesity and stress have been linked to an increased risk of atopic dermatitis (48, 49) and are at the same time more often observed in persons with nicotine dependence (49, 50). Therefore, being overweight and experiencing stress are potential confounding factors when determining the risk of nicotine dependence on any given outcome. Based on inconsistent published results on the risk for celiac disease imposed by nicotine dependence, Wijarnpreecha and colleagues performed a meta-analysis and found an almost 50% decreased risk of celiac disease among current smokers compared with never-smokers (24). This finding contrasts with the increased risk for celiac disease imposed by nicotine dependence reported here. Again, this meta-analysis did not consider potential confounding factors, e.g., obesity and stress, which are more common in patients with celiac disease compared to respective controls (51, 52). In an umbrella review of meta-analyses, nicotine dependence increased the risk for Crohn’s disease, but not ulcerative colitis (53). In contrast, other studies suggested that nicotine dependence confers protection for ulcerative colitis (54, 55). We here document that nicotine dependence increased the risk for both Crohn’s disease and ulcerative colitis. As already discussed in this paragraph, these differences may stem from the lack of or different matching strategies. The observed differences in the risk of developing either Crohn’s disease or ulcerative colitis may also be related to the status of nicotine dependence: Persons with a history of nicotine dependence have a higher risk for ulcerative colitis, but current nicotine dependence confers protection thereof (56, 57). Collectively, the impact of nicotine dependence on the risk for Crohn’s disease and ulcerative colitis remains, to an extent, uncertain because we here did not differentiate between current and past nicotine dependence. Overall, as shown here, we tend to assume that nicotine dependence is associated with an increased risk for both diseases.

Importantly, we here also shed light on the impact of nicotine dependence on the risk of developing CIDs where insights into this risk had been scant or not been determined. We furthermore provide insights into sex-specific risks imposed by nicotine dependence. Here, only slight differences were noted when analyzing the CID risks in EHRs indicating female or male sex.

These findings, however, do not allow to infer causality. Yet, a significant body of evidence points towards a causal relationship. More specifically, nicotine has been shown to induce a state of chronic low-grade inflammation, characterized by elevated levels of pro-inflammatory cytokines, which can potentially lead to the development of CIDs (58). Furthermore, nicotine’s effects on the immune system may alter the balance of pro-inflammatory and anti-inflammatory responses, thereby influencing disease progression in RA and asthma (59, 60). Additionally, nicotine dependence often coexists with other lifestyle factors, such as poor diet and lack of physical activity, which further heighten the risk for CIDs by promoting metabolic dysregulation and systemic inflammation (61).

The validity of our findings is underscored by replicating the previously documented increased risk of nicotine dependence for MI, lung cancer, COPD, SLE, RA, hidradenitis suppurativa, psoriasis, multiple sclerosis, and asthma (8, 10–15, 41, 42). However, this study has several limitations to be appreciated. First, owing to the retrospective design of the study, observed differences in CID risks cannot be causally attributed to underlying nicotine dependence. Second, the TriNetX database provides EHRs and is thus open to misdiagnosis or false coding of diagnoses and demographic information used for matching. This may also cause missing information in the EHRs, if, for example, nicotine dependence is not evaluated in subjects. As the data are not ascertained in a systematic manner, more specifically asking each patient regarding nicotine dependence, subjects in the unexposed group may have been exposed to nicotine dependence. If nicotine dependence is documented, the probability of this being correct is higher. We therefore decided to define nicotine dependence as current or past nicotine dependence to minimize the bias due to missing diagnostic codes. This will, however, lead to an underestimation, rather than an overestimation of the impact of nicotine dependence on CID risk. Next, the lack of differentiation between both forms of nicotine dependence does not allow us to discern between the risks of present or past nicotine dependence, which likely is important for some diseases, e.g., inflammatory bowel diseases (56, 57). In the same line, we decided to classify individuals only into unexposed or nicotine dependence (ICD10:F17), without specifying the type of nicotine consumption. Nicotine dependence includes ICD10 codes F17.20 “Nicotine dependence, unspecified” (*n* = 3.9 million), F17.21 “Nicotine dependence, cigarettes” (*n* = 2.6 million EHRS), F17.22 “Nicotine dependence, chewing tobacco” (*n* = 130,000 EHRs), and F17.29 “Nicotine dependence, other tobacco product” (*n* = 700,000 EHRs). This allows us to capture all forms of nicotine dependence but does not allow us to differentiate among the different forms of nicotine dependence. Hence, one may assume that in the majority of EHRs where nicotine dependence was indicated, nicotine was consumed by smoking. This also precludes any investigation on the risks imposed by consumption of nicotine by the use of electronic devices, which is rapidly growing among nonsmokers (62). Consumption of nicotine (and other compounds present when exposed to smoking or vaping) is being recognized as a potential health threat (63). Furthermore, disease scores or data on severity and progression are not provided. Moreover, as patient records are reported by healthcare providers, accessibility of healthcare services is a potential confounder. Yet, we herein have validated the pipeline by replicating previous findings on the risk of nicotine dependence on MI, lung cancer, and COPD.

In conclusion, we document that nicotine dependence is a risk factor to develop CIDs. On the level of individual CIDs, nicotine dependence is associated with an increased risk for most CIDs. Of note, we also document sex-specific risks for CID development. These insights provide an extensive overview of the risk for CIDs imposed by nicotine dependence. Based on the overall increased risk for CID manifestation in nicotine-dependent persons, our study urges the implementation of more stringent measures to prevent nicotine dependence and, thus, CID development.

## Data Availability

The original contributions presented in the study are included in the article/**Supplementary Material**. Further inquiries can be directed to the corresponding author.
