# Supplementary material for: Nicotine dependence is associated with an increased risk of developing chronic, non-communicable inflammatory disease: a large-scale retrospective cohort study
Source: Front Psychiatry. 2025 Feb 12;16:1429297. doi: 10.3389/fpsyt.2025.1429297 (PMC11860976; doi:10.3389/fpsyt.2025.1429297)
Supplement: Supplementary file 1 [file Table1.docx]

**Supplement Tables**

| **Outcome** | **Past or current nicotine dependence** | | | | **Cigarette smoking** | | | |
| --- | --- | --- | --- | --- | --- | --- | --- | --- |
|  | **Cases**  **% \| total n** | **Controls**  **% \| total n** | **HR (95%-CI)** | **p*** | **Cases**  **% \| total n** | **Controls**  **% \| total n** | **HR (95%-CI)** | **p*** |
| **Malignant neoplasms of the lung** | 1.91 \| 1,158^1^ | 0.34 \| 1,178 | 6.07  (6.48-6.93) | < 0.0001 | 2.17 \| 294 | 0.58 \| 299 | 6.34  (5.99-6.71) | < 0.0001 |
| **Myocardial infarction** | 4.20 \| 1,102 | 2.25 \| 1,156 | 2.25  (2.21-2.28) | < 0.0001 | 3.84 \| 277 | 2.37 \| 293 | 2.81  (2.73-2.91) | < 0.0001 |
| **COPD** | 8.32 \| 933 | 1.76 \| 1,143 | 5.83  (5.74-5.92) | < 0.0001 | 9.72 \| 204 | 2.58 \| 288 | 6.34  (6.17-6.52) | < 0.0001 |
| **Any CID** | 16.10 \| 653 | 10.99 \| 906 | 1.81  (1.80-1.83) | < 0.0001 | 15.34 \| 141 | 12.43 \| 223 | 2.03  (2.00-2.08) | < 0.0001 |
| **Any CID without asthma and COPD** | 7.21 \| 955 | 6.53 \| 1,035 | 1.32  (1.30-1.33) | < 0.0001 | 5.79 \| 242 | 7.17 \| 261 | 1.30  (1.27-1.33) | < 0.0001 |

**Supplement Table 1**. Similar risks for malignant neoplasms of the lung, myocardial infarction, COPD, any CID, and any CID without asthma and COPD in persons exposed to past or current nicotine dependence and in those exposed to cigarette smoking. Percentage indicates proportion of EHRs with the respective documented outcome. Total n relates to the ^1^number x 10^3^ of EHRs in cases and controls after propensity-score matching. ***(**alpha_adj_=0.0013)

| **Characteristic** | **Before matching** | | | **After matching** | | |
| --- | --- | --- | --- | --- | --- | --- |
|  | **Nicotine dependence (exposed)** | **Unexposed** | **P value** | **Nicotine dependence (exposed)** | **Unexposed** | **P value** |
| Number of participants | 422,610 | 3,683,27 | - | 414,765 | 414,765 | * |
| Age at Index | 56 ± 17.2 | 31.4 ± 25.8 | < 0.0001 | 55.9 ± 17.2 | 56 ± 17.3 | < 0.0001 |
| White | 72.275 | 60.93 | < 0.0001 | 72.079 | 72.016 | 0.5215 |
| Female | 100 | 100 | n.s. | 100 | 100 | n.s. |
| Overweight and obesity | 37.392 | 12.241 | < 0.0001 | 36.688 | 37.024 | 0.0015 |
| Reaction to severe stress, and adjustment disorders | 14.862 | 3.349 | < 0.0001 | 14.176 | 14.689 | < 0.0001 |
| Problems related to life management difficulty | 3.608 | 0.239 | < 0.0001 | 2.283 | 1.614 | < 0.0001 |
| Family history of other diseases of the musculoskeletal system and connective tissue | 0.318 | 0.086 | < 0.0001 | 0.294 | 0.272 | 0.0599 |

**Supplement Table 2**. Baseline characteristics before and after propensity-score matching of electronic medical records (EMRs) indicating current or past nicotine dependence (exposed) and those without the documentation of current of past nicotine dependence (unexposed) stratified for female sex.

| **Diagnosis** | **ICD10 code** | **Nicotine dependence (exposed)** | | | **Unexposed** | | |  |  |  |  |  |
| --- | --- | --- | --- | --- | --- | --- | --- | --- | --- | --- | --- | --- |
|  |  | **N of eligible partici-pants** | **N of Out-comes** | **Risk, %** | **N of eligible partici-pants** | **N of Out-comes** | **Risk, %** | **Risk difference, %** | **95% confidence interval** | **Hazard ratio** | **95% confidence interval** | **P value (adjusted α=0.0013)** |
| Acute myocardial infarction | I21 ***or*** I22 | 388,617 | 16,686 | 4.294 | 408,824 | 6,312 | 1.544 | 2.75 | (2.676,2.824) | 3.129 | (3.04,3.222) | < 0.0001 |
| Malignant neoplasm of lung and bronchus | C34 | 402,765 | 8,741 | 2.17 | 413,213 | 1,245 | 0.301 | 1.869 | (1.821,1.917) | 8.14 | (7.67,8.639) | < 0.0001 |
| **Any** | **Any of the below** | **206,807** | **36,938** | **17.861** | **307,295** | **33,338** | **10.849** | **7.012** | **(6.814,7.211)** | **1.913** | **(1.885,1.941)** | **< 0.0001** |
| ***Any, except J44 and J45*** |  | ***310,377*** | ***29,011*** | ***9.347*** | ***353,394*** | ***24,995*** | ***7.073*** | ***2.274*** | ***(2.141, 2.407)*** | ***1.48*** | ***(1.455,1.506)*** | ***< 0.0001*** |
| Other chronic obstructive pulmonary disease | J44 | 334,438 | 26,791 | 8.011 | 404,119 | 5,867 | 1.452 | 6.559 | (6.46,6.658) | 6.386 | (6.207,6.57) | < 0.0001 |
| Systemic lupus erythematosus | M32 | 407,825 | 1,755 | 0.43 | 411,195 | 1,013 | 0.246 | 0.184 | (0.159,0.209) | 1.92 | (1.777,2.075) | < 0.0001 |
| Systemic sclerosis | M34 | 412,963 | 613 | 0.148 | 413,915 | 355 | 0.086 | 0.063 | (0.048,0.077) | 1.903 | (1.669,2.17) | < 0.0001 |
| Rheumatoid arthritis | M05 ***or*** M06. | 390,222 | 7,506 | 1.924 | 403,512 | 4,322 | 1.071 | 0.852 | (0.799,0.906) | 1.985 | (1.912,2.061) | < 0.0001 |
| Sjögren syndrome | M35.0 | 408,745 | 3,345 | 0.818 | 410,466 | 3,400 | 0.828 | -0.01 | (-0.049,0.029) | 1.126 | (1.073,1.181) | < 0.0001 |
| Ankylosing spondylitis | M45 | 413,509 | 635 | 0.154 | 414,149 | 399 | 0.096 | 0.057 | (0.042,0.072) | 1.784 | (1.573,2.023) | < 0.0001 |
| Polyarteritis nodosa | M30.0 | 414,471 | 156 | 0.038 | 414,681 | 41 | 0.01 | 0.028 | (0.021,0.034) | 4.044 | (2.866,5.708) | < 0.0001 |
| Polymyalgia rheumatica | M35.3 | 411,781 | 1,177 | 0.286 | 412,729 | 1,193 | 0.289 | -0.003 | (-0.026,0.02) | 1.097 | (1.012,1.19) | 0.0245 |
| Dermatomyositis | M33 | 413,831 | 307 | 0.074 | 414,164 | 204 | 0.049 | 0.025 | (0.014,0.036) | 1.645 | (1.378,1.965) | < 0.0001 |
| Granulomatosis with polyangiitis | M31.3 | 414,348 | 211 | 0.051 | 414,608 | 64 | 0.015 | 0.035 | (0.028,0.043) | 3.641 | (2.75,4.821) | < 0.0001 |
| Eosinophilc granulomatosis with polyangiitis | M30.1 | 414,585 | 136 | 0.033 | 414,723 | 29 | 0.007 | 0.026 | (0.02,0.032) | 5.038 | (3.371,7.529) | < 0.0001 |
| Sarcoidosis | D86 | 411,493 | 955 | 0.232 | 412,753 | 619 | 0.15 | 0.082 | (0.063,0.101) | 1.7 | (1.536,1.881) | < 0.0001 |
| Pyoderma gangrenosum | L88 | 414,490 | 170 | 0.041 | 414,683 | 60 | 0.014 | 0.027 | (0.019,0.034) | 3.121 | (2.323,4.194) | < 0.0001 |
| Hidradenitis suppurativa | L73.2 | 408,223 | 2,941 | 0.72 | 412,926 | 1,113 | 0.27 | 0.451 | (0.421,0.481) | 2.972 | (2.774,3.185) | < 0.0001 |
| Alopecia areata | L63 | 413,203 | 665 | 0.161 | 413,846 | 577 | 0.139 | 0.022 | (0.005,0.038) | 1.293 | (1.156,1.446) | < 0.0001 |
| Vitiligo | L80 | 412,815 | 829 | 0.201 | 413,544 | 656 | 0.159 | 0.042 | (0.024,0.06) | 1.397 | (1.261,1.549) | < 0.0001 |
| Psoriasis vulgaris | L40.0 | 409,077 | 1,429 | 0.349 | 411,725 | 1,153 | 0.28 | 0.069 | (0.045,0.094) | 1.37 | (1.267,1.481) | < 0.0001 |
| Atopic dermatitis | L20 | 392,900 | 4,758 | 1.211 | 397,818 | 3,879 | 0.975 | 0.236 | (0.19,0.282) | 1.364 | (1.307,1.423) | < 0.0001 |
| Lichen planus | L43 | 412,351 | 1,251 | 0.303 | 413,418 | 1,134 | 0.274 | 0.029 | (0.006,0.052) | 1.24 | (1.144,1.344) | < 0.0001 |
| Morphea | L94.0 | 412,842 | 518 | 0.125 | 413,365 | 740 | 0.179 | -0.054 | (-0.07,-0.037) | 0.77 | (0.688,0.861) | < 0.0001 |
| Pemphigus (PV and PF) | L10.0 ***or*** L10.1 ***or*** L10.2 ***or*** L10.4 | 414,549 | 139 | 0.034 | 414,645 | 65 | 0.016 | 0.018 | (0.011,0.025) | 2.339 | (1.74,3.143) | < 0.0001 |
| Bullous pemphigoid | L12.0 | 414,529 | 172 | 0.041 | 414,626 | 126 | 0.03 | 0.011 | (0.003,0.019) | 1.554 | (1.234,1.958) | 0.0002 |
| Mucous membrane pemphigoid | L12.1 | 414,670 | 35 | 0.008 | 414,701 | 27 | 0.007 | 0.002 | (-0.002,0.006) | 1.458 | (0.881,2.414) | 0.1404 |
| Psoriatic arthritis | L40.5 | 411,783 | 1,184 | 0.288 | 413,143 | 748 | 0.181 | 0.106 | (0.086,0.127) | 1.77 | (1.614,1.94) | < 0.0001 |
| Lichen sclerosus | L90.0 | 411,212 | 1,830 | 0.445 | 412,236 | 2,150 | 0.522 | -0.077 | (-0.106,-0.047) | 0.955 | (0.897,1.017) | 0.1504 |
| Celiac disease | K90.0 | 410,349 | 1,704 | 0.415 | 412,423 | 918 | 0.223 | 0.193 | (0.168,0.217) | 2.042 | (1.884,2.213) | < 0.0001 |
| Crohn's disease | K50 | 408,750 | 1,906 | 0.466 | 412,322 | 779 | 0.189 | 0.277 | (0.253,0.302) | 2.725 | (2.506,2.962) | < 0.0001 |
| Ulcerative colitis | K51 | 409,296 | 2,609 | 0.637 | 411,786 | 1,705 | 0.414 | 0.223 | (0.192,0.255) | 1.717 | (1.615,1.825) | < 0.0001 |
| Primary biliary cirrhosis | K74.3 | 402,418 | 433 | 0.108 | 402,907 | 170 | 0.042 | 0.065 | (0.053,0.077) | 2.826 | (2.366,3.377) | < 0.0001 |
| Autoimmune hepatitis | K75.4 | 402,294 | 538 | 0.134 | 402,868 | 215 | 0.053 | 0.08 | (0.067,0.094) | 2.736 | (2.335,3.207) | < 0.0001 |
| Autoimmune thyroiditis | E06.3 | 395,649 | 2,936 | 0.742 | 396,850 | 3,482 | 0.877 | -0.135 | (-0.175,-0.096) | 0.92 | (0.875,0.966) | 0.0008 |
| Diabetes type 1 | E10 | 388,948 | 4,325 | 1.112 | 395,554 | 2,401 | 0.607 | 0.505 | (0.464,0.546) | 1.994 | (1.897,2.097) | < 0.0001 |
| Vitamin B12 deficiency anemia due to intrinsic factor deficiency | D51.0 | 400,845 | 935 | 0.233 | 401,705 | 837 | 0.208 | 0.025 | (0.004,0.045) | 1.22 | (1.111,1.339) | < 0.0001 |
| Other autoimmune hemolytic anemia | D59.1 | 402,110 | 871 | 0.217 | 403,084 | 228 | 0.057 | 0.16 | (0.144,0.176) | 4.208 | (3.636,4.87) | < 0.0001 |
| Immune thrombocytopenic purpura | D69.3 | 402,267 | 460 | 0.114 | 402,748 | 381 | 0.095 | 0.02 | (0.006,0.034) | 1.324 | (1.156,1.518) | < 0.0001 |
| Myasthenia gravis | G70 | 401,355 | 1,370 | 0.341 | 402,55 | 608 | 0.151 | 0.19 | (0.169,0.212) | 2.476 | (2.25,2.725) | < 0.0001 |
| Multiple sclerosis | G35 | 398,070 | 1,861 | 0.468 | 400,743 | 644 | 0.161 | 0.307 | (0.282,0.331) | 3.192 | (2.917,3.492) | < 0.0001 |
| Asthma | J45 | 303,971 | 19,795 | 6.512 | 350,442 | 15,297 | 4.365 | 2.147 | (2.036,2.258) | 1.656 | (1.622,1.692) | < 0.0001 |

**Supplement Table 3**. Impact of nicotine dependence on the risk to develop chronic, non-communicable inflammatory diseases stratified for female sex. Exposed individuals (with history of current or past nicotine dependence) were matched 1:1 to unexposed individuals (without any documentation of current of past nicotine dependence at the index health care visit) using age, ethnicity, and risk factors of chronic, non-communicable inflammatory diseases. Non-significant data (after adjustment for multiple testing) is indicated by light grey letters. Hazard ratios were calculated by univariate Cox regression P values were determined by the Log-rank test.

| **Characteristic** | **Before matching** | | | **After matching** | | |
| --- | --- | --- | --- | --- | --- | --- |
|  | **Nicotine dependence (exposed)** | **Unexposed** | **P value** | **Nicotine dependence (exposed)** | **Unexposed** | **P value** |
| Number of participants | 383,600 | 3,034,059 | - | 373,006 | 373,006 | - |
| Age at Index | 58.8 ± 16.6 | 26.5 ± 25.5 | < 0.0001 | 58.5 ± 16.6 | 58.6 ± 16.7 | 0.0012 |
| White | 74.507 | 61.206 | < 0.0001 | 74.246 | 73.927 | 0.0017 |
| Female | 0 | 0 | n.s. | 0 | 0 | n.s. |
| Overweight and obesity | 31.433 | 9.029 | < 0.0001 | 30.159 | 30.107 | 0.6262 |
| Reaction to severe stress, and adjustment disorders | 8.164 | 1.779 | < 0.0001 | 7.215 | 7.488 | < 0.0001 |
| Problems related to life management difficulty | 2.336 | 0.177 | < 0.0001 | 1.236 | 0.83 | < 0.0001 |
| Family history of other diseases of the musculoskeletal system and connective tissue | 0.144 | 0.048 | < 0.0001 | 0.122 | 0.111 | 0.1748 |

**Supplement Table 4**. Baseline characteristics before and after propensity-score matching of electronic medical records (EMRs) indicating current or past nicotine dependence (exposed) and those without the documentation of current of past nicotine dependence (unexposed) stratified for male sex.

| **Diagnosis** | **ICD10 code** | **Nicotine dependence (exposed)** | | | **Unexposed** | | |  |  |  |  |  |
| --- | --- | --- | --- | --- | --- | --- | --- | --- | --- | --- | --- | --- |
|  |  | **N of eligible partici-pants** | **N of Out-comes** | **Risk, %** | **N of eligible partici-pants** | **N of Out-comes** | **Risk, %** | **Risk difference, %** | **95% confidence interval** | **Hazard ratio** | **95% confidence interval** | **P value (adjusted α=0.0013)** |
| Acute myocardial infarction | I21 ***or*** I22 | 337,403 | 17,987 | 5.331 | 363,821 | 7,780 | 2.138 | 3.193 | (3.103,3.282) | 2.903 | (2.826,2.982) | < 0.0001 |
| Malignant neoplasm of lung and bronchus | C34 | 363,067 | 7,990 | 2.201 | 371,670 | 1,284 | 0.345 | 1.855 | (1.804,1.907) | 7.4 | (6.975,7.85) | < 0.0001 |
| **Any** | **Any of the below** | **225,857** | **31,978** | **14.159** | **307,814** | **23,343** | **7.583** | **6.575** | **(6.404,6.747)** | **2.219** | **(2.182,2.257)** | **< 0.0001** |
| ***Any, except J44 and J45*** |  | ***296,444*** | ***19,395*** | ***6.543*** | ***325,074*** | ***14,705*** | ***4.524*** | ***4.524*** | ***1.905, 2.133*** | ***1.657*** | ***(1.622,1.693)*** | ***< 0.0001*** |
| Other chronic obstructive pulmonary disease | J44 | 302,724 | 23,163 | 7.652 | 363,026 | 5,090 | 1.402 | 6.249 | (6.147,6.352) | 6.472 | (6.278,6.672) | < 0.0001 |
| Systemic lupus erythematosus | M32 | 371,590 | 496 | 0.133 | 372,542 | 184 | 0.049 | 0.084 | (0.07,0.098) | 3.017 | (2.545,3.575) | < 0.0001 |
| Systemic sclerosis | M34 | 372,546 | 240 | 0.064 | 372,860 | 92 | 0.025 | 0.04 | (0.03,0.049) | 2.914 | (2.289,3.709) | < 0.0001 |
| Rheumatoid arthritis | M05 ***or*** M06. | 360,995 | 4,125 | 1.143 | 368,742 | 1,959 | 0.531 | 0.611 | (0.57,0.653) | 2.443 | (2.314,2.578) | < 0.0001 |
| Sjögren syndrome | M35.0 | 371,824 | 1,402 | 0.28 | 372,288 | 999 | 0.268 | 0.012 | (-0.012,0.036) | 1.263 | (1.157,1.378) | < 0.0001 |
| Ankylosing spondylitis | M45 | 371,625 | 512 | 0.138 | 372,276 | 342 | 0.092 | 0.046 | (0.031,0.061) | 1.707 | (1.488,1.959) | < 0.0001 |
| Polyarteritis nodosa | M30.0 | 372,787 | 111 | 0.03 | 372,955 | 24 | 0.006 | 0.023 | (0.017,0.029) | 5.035 | (3.236,7.836) | < 0.0001 |
| Polymyalgia rheumatica | M35.3 | 370,999 | 878 | 0.237 | 371,599 | 991 | 0.267 | -0.03 | (-0.053,0.007) | 1.021 | (0.932,1.118) | 0.6607 |
| Dermatomyositis | M33 | 372,479 | 163 | 0.044 | 372,702 | 136 | 0.036 | 0.007 | (-0.002,0.016) | 1.313 | (1.044,1.65) | 0.0193 |
| Granulomatosis with polyangiitis | M31.3 | 372,657 | 186 | 0.05 | 372,885 | 41 | 0.011 | 0.039 | (0.031,0.047) | 5.014 | (3.571,7.04) | < 0.0001 |
| Eosinophilc granulomatosis with polyangiitis | M30.1 | 372,883 | 91 | 0.024 | 372,988 | 21 | 0.006 | 0.019 | (0.013,0.024) | 4.694 | (2.918,7.551) | < 0.0001 |
| Sarcoidosis | D86 | 370,998 | 661 | 0.178 | 371,645 | 469 | 0.126 | 0.052 | (0.034,0.07) | 1.592 | (1.414,1.793) | < 0.0001 |
| Pyoderma gangrenosum | L88 | 372,871 | 87 | 0.023 | 372,952 | 35 | 0.009 | 0.014 | (0.008,0.02) | 2.907 | (1.958,4.315) | < 0.0001 |
| Hidradenitis suppurativa | L73.2 | 370,980 | 881 | 0.237 | 372,496 | 330 | 0.089 | 0.149 | (0.131,0.167) | 3.045 | (2.681,3.457) | < 0.0001 |
| Alopecia areata | L63 | 372,323 | 274 | 0.074 | 372,577 | 245 | 0.066 | 0.008 | (-0.004,0.02) | 1.259 | (1.058,1.497) | 0.0091 |
| Vitiligo | L80 | 371,587 | 582 | 0.157 | 372,038 | 546 | 0.147 | 0.01 | (-0.008,0.028) | 1.19 | (1.058,1.338) | 0.0037 |
| Psoriasis vulgaris | L40.0 | 367,706 | 1,140 | 0.31 | 369,941 | 1,024 | 0.277 | 0.033 | (0.009,0.058) | 1.248 | (1.147,1.359) | < 0.0001 |
| Atopic dermatitis | L20 | 365,620 | 3,381 | 0.925 | 369,279 | 2,620 | 0.709 | 0.215 | (0.174,0.26) | 1.485 | (1.41,1.563) | < 0.0001 |
| Lichen planus | L43 | 371,691 | 661 | 0.178 | 372,327 | 547 | 0.147 | 0.031 | (0.013,0.049) | 1.406 | (1.254,1.576) | < 0.0001 |
| Morphea | L94.0 | 372,736 | 83 | 0.022 | 372,817 | 86 | 0.023 | -0.001 | (-0.008,0.006) | 1.12 | (0.826,1.517) | 0.4659 |
| Pemphigus (PV and PF) | L10.0 ***or*** L10.1 ***or*** L10.2 ***or*** L10.4 | 372,812 | 106 | 0.028 | 372,884 | 49 | 0.013 | 0.015 | (0.009,0.022) | 2.449 | (1.742,3.443) | < 0.0001 |
| Bullous pemphigoid | L12.0 | 372,715 | 196 | 0.053 | 372,860 | 143 | 0.038 | 0.014 | (0.005,0.024) | 1.595 | (1.284,1.981) | < 0.0001 |
| Mucous membrane pemphigoid | L12.1 | 372,917 | 23 | 0.006 | 372,945 | 22 | 0.006 | 0 | (-0.003,0.004) | 1.209 | (0.672,2.177) | 0.5262 |
| Psoriatic arthritis | L40.5 | 370,822 | 688 | 0.186 | 371,566 | 506 | 0.136 | 0.049 | (0.031,0.068) | 1.547 | (1.379,1.737) | < 0.0001 |
| Lichen sclerosus | L90.0 | 372,711 | 119 | 0.032 | 372,817 | 111 | 0.03 | 0.002 | (-0.006,0.01) | 1.226 | (0.945,1.59) | 0.1240 |
| Celiac disease | K90.0 | 371,228 | 741 | 0.2 | 371,978 | 446 | 0.12 | 0.08 | (0.062,0.098) | 1.874 | (1.665,2.109) | < 0.0001 |
| Crohn's disease | K50 | 368,640 | 1,315 | 0.357 | 371,065 | 584 | 0.157 | 0.199 | (0.176,0.222) | 2.552 | (2.314,2.814) | < 0.0001 |
| Ulcerative colitis | K51 | 368,302 | 2,166 | 0.588 | 370,271 | 1,527 | 0.412 | 0.176 | (0.144,0.208) | 1.647 | (1.542,1.759) | < 0.0001 |
| Primary biliary cirrhosis | K74.3 | 372,644 | 171 | 0.046 | 372,876 | 72 | 0.019 | 0.027 | (0.018,0.035) | 2.696 | (2.044,3.556) | < 0.0001 |
| Autoimmune hepatitis | K75.4 | 372,536 | 292 | 0.078 | 372,846 | 90 | 0.024 | 0.054 | (0.044,0.065) | 3.623 | (2.858,4.592) | < 0.0001 |
| Autoimmune thyroiditis | E06.3 | 371,209 | 848 | 0.228 | 371,710 | 829 | 0.223 | 0.005 | (-0.016,0.027) | 1.143 | (1.038,1.258) | 0.0065 |
| Diabetes type 1 | E10 | 356,008 | 4,888 | 1.373 | 364,349 | 3,047 | 0.836 | 0.537 | (0.488,0.585) | 1.846 | (1.764,1.932) | < 0.0001 |
| Vitamin B12 deficiency anemia due to intrinsic factor deficiency | D51.0 | 371,606 | 579 | 0.156 | 371,963 | 532 | 0.143 | 0.013 | (-0.005,0.03) | 1.22 | (1.084,1.373) | 0.0010 |
| Other autoimmune hemolytic anemia | D59.1 | 371,827 | 808 | 0.217 | 372,785 | 195 | 0.052 | 0.165 | (0.148,0.182) | 4.686 | (4.006,5.483) | < 0.0001 |
| Immune thrombocytopenic purpura | D69.3 | 371,918 | 566 | 0.152 | 372,425 | 408 | 0.11 | 0.043 | (0.026,0.059) | 1.582 | (1.392,1.798) | < 0.0001 |
| Myasthenia gravis | G70 | 371,092 | 1,559 | 0.42 | 372,129 | 756 | 0.203 | 0.217 | (0.192,0.242) | 2.365 | (2.167,2.58) | < 0.0001 |
| Multiple sclerosis | G35 | 370,732 | 935 | 0.252 | 372,045 | 253 | 0.068 | 0.184 | (0.166,0.202) | 4.19 | (3.645,4.817) | < 0.0001 |
| Asthma | J45 | 318,612 | 12,982 | 4.075 | 345,119 | 9,035 | 2.618 | 1.457 | (1.37,1.544) | 1.758 | (1.711,1.806) | < 0.0001 |

**Supplement Table 5**. Impact of nicotine dependence on the risk to develop chronic, non-communicable inflammatory diseases stratified for male sex. Exposed individuals (with history of current or past nicotine dependence) were matched 1:1 to unexposed individuals (without any documentation of current of past nicotine dependence at the index health care visit) using age, ethnicity, and risk factors of chronic, non-communicable inflammatory diseases. Non-significant data (after adjustment for multiple testing) is indicated by light grey letters. Hazard ratios were calculated by univariate Cox regression P values were determined by the Log-rank test.
