# Supplementary figures and images for: Nicotine dependence is associated with an increased risk of developing chronic, non-communicable inflammatory disease: a large-scale retrospective cohort study
Source: Front Psychiatry. 2025 Feb 12;16:1429297. doi: 10.3389/fpsyt.2025.1429297 (PMC11860976; doi:10.3389/fpsyt.2025.1429297)

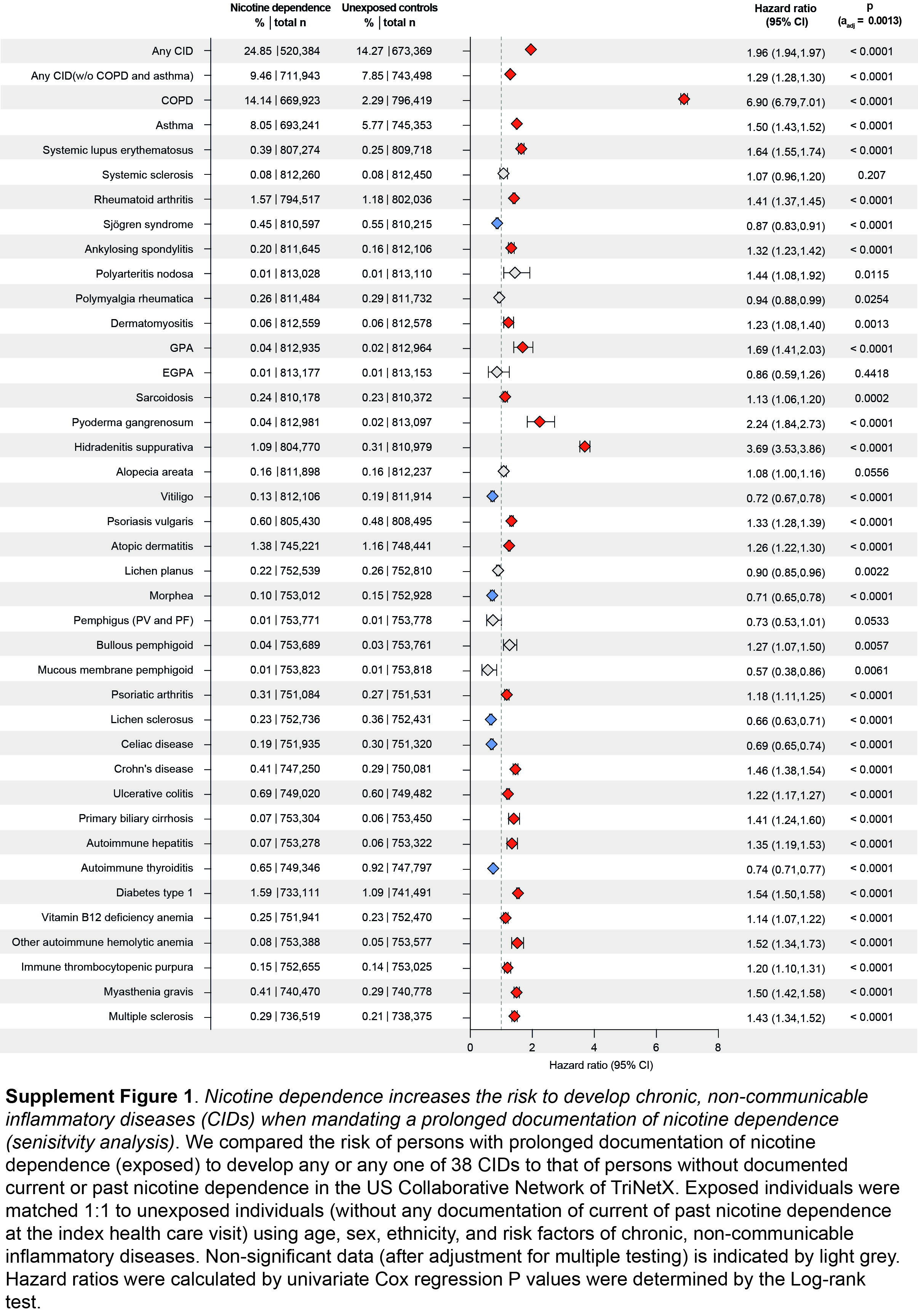

Supplement: Supplementary file 2 [file Image1.jpeg]
